# Combination of PD-L1 expression and NLR as prognostic marker in patients with surgically resected non-small cell lung cancer

**DOI:** 10.7150/jca.34469

**Published:** 2019-10-22

**Authors:** Xinyue Wang, Lianjing Cao, Shouying Li, Fan Wang, Dingzhi Huang, Richeng Jiang

**Affiliations:** 1 Tianjin Medical University Cancer Institute & Hospital, National Clinical Research Center for Cancer; 2 Key Laboratory of Cancer Prevention and Therapy, Tianjin; 3 Tianjin's Clinical Research Center for Cancer; 4 Department of Thoracic Oncology, Tianjin Lung Cancer Center, Tianjin Cancer Institute & Hospital, Tianjin Medical University, Tianjin 300060, PR China; 5 State Key Laboratory of Oncology in South China, Collaborative Innovation Center for Cancer Medicine, Sun Yat-sen University Cancer Center, Guangzhou 510060, China

**Keywords:** neutrophil-lymphocyte ratio, programmed death ligand-1, immunohistochemical analysis, prognostic marker, non-small cell lung cancer

## Abstract

**Background:** In recent years, great improvement has been made in immunotherapies for non-small cell lung cancer (NSCLC). Current data have suggested that Programmed cell death ligand 1 (PD-L1) expression might not be an ideal marker for patient selection in isolation. Evidence has been increasing that alternative markers, such as neutrophil-to-lymphocyte ratio (NLR), a biomarker of systemic inflammation response (SIR) previously associated with outcomes in a variety of cancers including NSCLC, might be a predictor for patient selection and the response to therapy. No reports have examined the prognostic value of combination of PD-L1 expression and inflammatory markers such as NLR in NSCLC. This retrospective study explores the relationship between NLR and PD-L1 expression in NSCLC as well as the prognostic value of combination of PD-L1 expression and NLR.

**Method:** We evaluated tumor PD-L1 expression in 235 surgically resected NSCLC cases by immunohistochemical analysis. Carcinoma cells showing membranous staining for PD-L1 were considered PD-L1-positive cells (Figure 1). Cases with ≥1% tumor membrane staining were considered PD-L1-positive. The association of clinicopathological characteristics with PD-L1 expression was assessed by univariate and multivariate analyses. Moreover, univariate and multivariate analyses were performed to evaluate the predictive impact of PD-L1 expression and other factors on disease-free survival (DFS) and overall survival (OS).

**Result:** PD-L1 protein expression was elevated in 34.0% of patients at cut-off value of 1%. Univariate analyses showed that PD-L1 expression was significantly higher in men (χ^2^ =5.226, *P=*0.030), heavy smokers (χ^2^ =18.650, *P*<0.001), and patients with squamous cell carcinoma (χ^2^ =4.036, *P=*0.045). No correlations were noted between PD-L1 expression and age, EGFR mutation status or clinical stage. No significant correlations between PD-L1 protein expression and NLR were found. Multivariate logistic regression revealed that smoking index ≥400 was independent predictor of PD-L1 expression (odds ratio [OR], 3.375; *P* < 0.001). The results of univariate survival analyses showed that clinical stage (log-rank χ^2^ =7.876, *P*=0.019) was associated with DFS. Smoking index (log-rank χ^2^ =4.832, *P*=0.028), clinical stage (log-rank χ^2^ =7.582, *P*=0.023) and adjuvant treatment (log-rank χ^2^ =5.440, *P*=0.020) were significantly associated with OS. Neither PD-L1 expression nor NLR was found to be associated with DFS or OS. Of interest, when patients were divided in two groups according to combined PD-L1/NLR: patients with PD-L1+/ high NLR as Group 1, other patients as Group 2, Group 1 had significantly shorter DFS as well as OS than Group 2 (DFS: log-rank χ^2^ =5.231, *P*=0.022, Figure 2A; OS: log-rank χ^2^ =4.742, *P*=0.029, Figure 2B). In the multivariate analysis, Cox proportional hazards regression models showed that, PD-L1+/ high NLR was associated with a significantly shorter DFS and OS (hazard ratio [HR], 1.394, *P*=0.040; HR, 1.442, *P*=0.042, respectively). Stratified analysis showed that the prognostic value of combined PD-L1/NLR can only be observed in cases without epidermal growth factor receptor (EGFR) mutations (DFS: log-rank χ^2^ =5.593, *P*=0.018, Figure 2C, OS: log-rank χ^2^ =9.323, *P*=0.002, Figure 2D). In EGFR mutation subgroup, combination of PD-L1 expression and NLR has no relationship with DFS or OS.

**Conclusion:** We found that combination of PD-L1 expression and NLR may be a promising prognostic indicator, and may also be a good marker for tumor recurrence, especially in the patients with wild-type EGFR.

## Introduction

Lung cancer is currently the top cause of cancer deaths worldwide 1. Non-small cell lung cancer (NSCLC), which accounts for more than 80% of all lung cancers, usually presents in a late stage in approximately 80% of cases. Despite rapid progress in the diagnosis and treatment of lung cancer, its prognosis remains poor, with 5-year survival rates of less than 15%. In recent years, great improvement has been made in immunotherapies for NSCLC, particularly monoclonal antibodies targeting the cytotoxic T-lymphocyte-associated protein 4 (CTLA-4), programmed death-1(PD-1) and its ligand (PD-L1). Although immune checkpoint inhibitors appear capable of producing durable responses compared to existing treatments, unfortunately a substantial proportion of patients treated with immune checkpoint inhibitors do not respond^2^, ^3^.

Accordingly, the identification of biomarkers that predict the clinical efficacy of immune checkpoint blockade therapy is urgent. Furthermore, effective combined therapies with immunotherapy are required for improved clinical benefit. Current data have indicated that the correlation between PD-L1 expression by immunohistochemistry and the response to immune checkpoint inhibition varies by both tumors histologic features, suggesting that PD-L1 might not be an ideal marker for patient selection in isolation 4. Nonetheless, evidence has been increasing that alternative markers, such as neutrophil-to-lymphocyte ratio (NLR), a biomarker of systemic inflammation response (SIR) previously associated with outcomes in a variety of cancers including NSCLC^5^-^12^, might be a predictor for patient selection and the response to therapy. High NLR has been reported to be associated with poor prognosis in patients with advanced NSCLC that were receiving immunotherapies^13^, ^14^. However, no reports have examined the potential prognostic value of combination of PD-L1 expression and inflammatory markers such as NLR. Herein, this retrospective study explores the relationship between NLR and PD-L1 expression in NSCLC as well as the prognostic value of combination of PD-L1 expression and NLR. Elucidation of the clinical significance of PD-L1 protein expression in combine with inflammatory marker in NSCLC may provide insights into patient selection and effective strategies for PD-1/PD-L1 inhibitory treatment.

## Material & Methods

### Patients

Patients who underwent surgery for primary adenocarcinoma (AD) and squamous cell carcinoma (SCC) between 2012 and 2015 were identified from a retrospective review of a prospectively maintained database at Tianjin Cancer Institute & Hospital, Tianjin Medical University, Tianjin P.R. China. We excluded patients: (1) who were lost to follow up, (2) without R0 resections, (3) with distant metastases, (4) had insufficient laboratory data, (5) had clinical evidence of infection or other inflammatory conditions, (6) had received preoperative chemotherapy or irradiation, (7) whose matched paraffin embedded formalin-fixed (FFPE) tissue specimens were unavailable. Finally, 235 patients entered our study. Information about demographics, data regarding surgical procedures, preoperative blood variables, postoperative course, pathologic findings, and follow-up was collected. The NLR was defined as the absolute neutrophil count divided by the absolute lymphocyte count. DFS and OS were stratified by median of NLR (2.3), which was also consisting with previous studies 15-18 . Epidermal growth factor receptor (EGFR) mutation status was detected by real-time PCR or DNA sequencing as previously described 19.

All Patients were observed until death or July 1, 2017. The median follow-up period was 35 months. Disease-free survival (DFS) was defined as the time from resection to the first disease recurrence and was censored at the last follow-up date if no events had occurred. Overall survival (OS) was calculated from the date of surgery to the date of death or last follow-up. Prior consent from all patients and approval from the Research Ethics Committee of Tianjin University were obtained for the use of clinical and pathological data.

## Immunohistochemistry for PD-L1

Tumor PD-L1 expression was evaluated by immunohistochemistry on formalin-fixed and paraffin embedded tumor tissue sections according to the previously described PD-L1 immunohistochemistry protocol 19. Briefly, formalin-fixed tissue sections were dewaxed with xylene followed by rehydrated through a graded series of ethanol and washed in distilled water (dH_2_O). Antigen retrieval was performed with EDTA buffer per the manufacture's, then sections were incubated in 3% hydrogen peroxide (H_2_O_2_) for 10min in order to inhibit endogenous peroxidase activity. The sections were incubated overnight at 4℃ with the monoclonal antibodies at 4°C overnight. To visualize the antigen, the immune complex was detected with a DAKO EnVision Detection System (Dako). Finally, sections were then counterstained with hematoxylin, and mounted. The primary antibody was an anti-human PD-L1 rabbit monoclonal antibody (rabbit anti-PD-L1 XP^®^ mAb 1:100, E1L3N, cell signaling Technology, Danvers, MA, United States of America (USA)). Carcinoma cells showing membranous staining for PD-L1 were considered PD-L1-positive cells. The proportion of PD-L1-positive cells was independently estimated as the percentage of total carcinoma cells in whole sections by two investigators (X.W. and L.C.). Cases with ≥1% tumor membrane staining were considered PD-L1-positive.

### Statistical Analysis

Categorical variables are presented as numbers and percentages. Distribution of continuous variables is reported as median and range. Qualitative data were compared by the χ^2^ test or Fisher's exact test when necessary. The logistic regression model was used to estimate influence of preoperative factors on PD-L1 expression. Survival probability was estimated by the Kaplan-Meier method, and log-rank test was used for the comparison of survival. Multivariate analyses were performed using the Cox proportional hazards regression model to evaluate significant recurrence predictors and prognostic factors. All tests were 2-sided. Statistical analyses were performed in SPSS 17.0 for Windows software (SPSS Inc). *P* values of <0.05 were considered to indicate statistical significance.

## Results

### Association between PD-L1 expression and clinicopathological characteristics

The clinicopathological characteristics of the 235 patients with NSCLC (130 with AD and 105 with SCC) are summarized in Table 1. The median age was 59 years (range 32-78). One hundred and fifty-three (65.1%) patients were male and 93 (39.6%) were heavy smokers (smoking index ≥ 400). Tumors of stages I, II, and III were observed in 112 (47.7%), 45 (19.1%) and 78 (33.2%) cases, respectively. Post-operative therapy was performed in 94 patients: 88 patients received chemotherapy; 3 were exposed to EGFR-TKI targeted therapy, 3 received radiation therapy, and 5 received both chemotherapy and radiotherapy. EGFR-mutation status was present in 48 patients (20.4%) and the other 187 (79.6%) cases had EGFR wild-type tumors.

**Figure 1:**
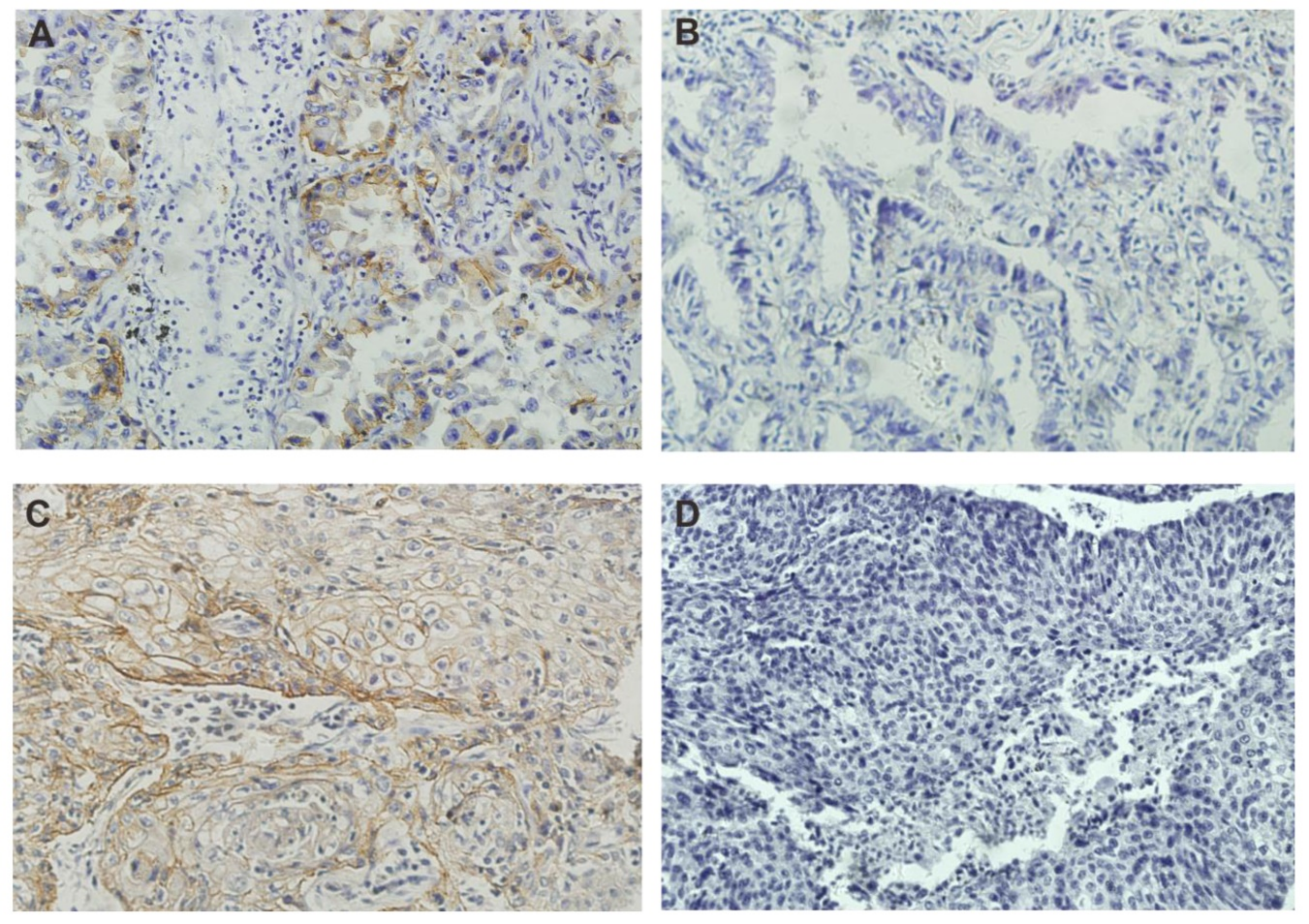
Expression of PD-L1 in lung adenocarcinomas (A, B) and squamous cell carcinomas (C, D). Representative images of PD-L1positive expression (A, C) and negative expression (B, D). Magnification ×200.

Immunohistochemical staining for PD-L1 was detected at the membrane of tumor cells (Figure 1). Eighty (34.0%) patients were positive for PD-L1 at the 1% cut-off value. The associations between PD-L1 expression and the clinicopathological features of the patients are summarized in Table 1. PD-L1 expression was significantly higher in men, heavy smokers, and patients with squamous cell carcinoma. No correlations were noted between PD-L1 expression and age, EGFR mutation status or clinical stage. Eighty (34.0%) patients had an NLR≥2.3. No significant correlations between PD-L1 protein expression and NLR were found. In a multivariable logistic regression analysis, smoking index ≥400 (odds ratio [OR], 3.375; 95% CI, 1.922-5.926; *P* < 0.001), was found to be independently associated with PD-L1 expression.

### Univariate and Multivariate Survival Analyses in All Patients

**Figure 2:**
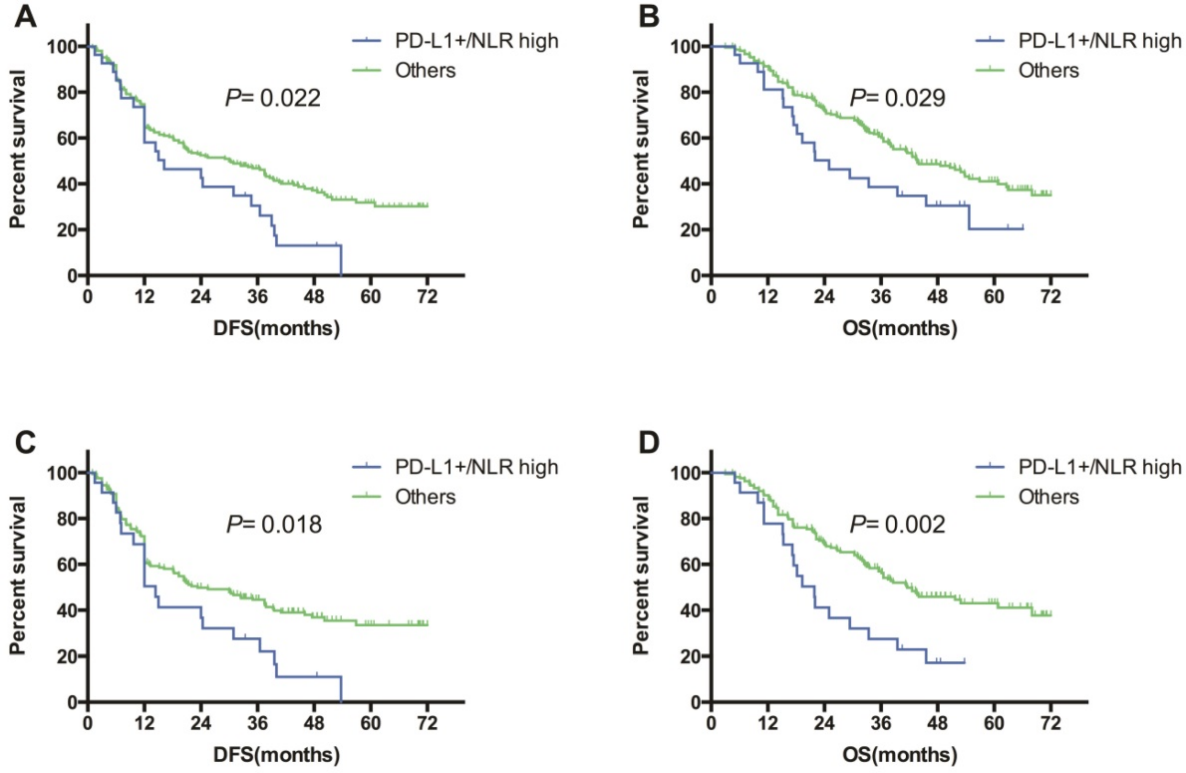
Kaplan-Meier survival curves of DFS (A, C) and OS (B, D) based on combination of PD-L1 expression and NLR (PD-L1+/ NLR high vs. others) in all patients (A, B), and in patients with wild-type EGFR(C, D).

The median follow-up period was 36.9 months. During the observation period, 132 (56.2%) patients died. Univariate and multivariate analyses were performed to evaluate the predictive impact of PD-L1 expression and other clinicopathological factors on OS and DFS (Table 2). The results of univariate analyses showed that clinical stage (log-rank χ^2^ =7.876, *P*=0.019) was associated with DFS. Smoking index (log-rank χ^2^ =4.832, *P*=0.028), clinical stage (log-rank χ^2^ =7.582, *P*=0.023) and adjuvant treatment (log-rank χ^2^ =5.440, *P*=0.020) were significantly associated with OS. Neither PD-L1 expression nor NLR was found to be associated with DFS or OS. Of interest, when patients were divided in two groups according to combined PD-L1/NLR: patients with PD-L1+/ high NLR as Group 1, other patients as Group 2, DFS and OS showed significant difference between groups. Group 1 had significantly shorter DFS as well as OS than Group 2 (DFS: log-rank χ^2^ =5.231, *P*=0.022, Figure 2A; OS: log-rank χ^2^ =4.742, *P*=0.029, Figure 2B).

In the multivariate analysis, Cox proportional hazards regression models showed that stage III and PD-L1+/ high NLR were associated with a significantly shorter DFS (stage III vs. stage I-II, HR, 1.559, *P*=0.009; Group 1 vs. Group 2: HR, 1.394, *P*=0.040, respectively). Moreover, clinical stage, smoking index, adjuvant treatment, and combined PD-L1/NLR remained independent factors of OS (stage III vs. stage I-II, HR, 1.831, *P*=0.001; SI≥400 vs. SI<400, HR, 1.616, *P*=0.008; with vs. without adjuvant treatment, HR, 0.544, *P*=0.001; Group 1 vs. Group 2: HR, 1.442, *P*=0.042, respectively, Table 3).

### DFS and OS According to NLR/PD-L1 in Subgroups

Subgroup analyses were performed for EGFR mutation status and adjuvant treatment (Table 2). In EGFR mutation subgroup, the results of wild-type subgroup analysis were similar with the results of entire cohort analysis that mentioned above: neither PD-L1 expression nor NLR was found to be associated with DFS or OS, PD-L1+/ high NLR was found to be associated with worse DFS (log-rank χ^2^ =5.593, *P* = 0.018, Figure 2C) and OS (log-rank χ^2^ =9.323, *P*=0.002, Figure 2D). However, in EGFR mutation subgroup, combination of PD-L1 expression and NLR has no relationship with DFS or OS. In addition, when patients were stratified according to adjuvant therapy, PD-L1+/ high NLR was associated with inferior OS (log-rank χ^2^=5.386, *P*=0.021) and a trend for worse DFS (log-rank χ^2^ =1.688, *P*=0.194) in patients without adjuvant chemotherapies. In patients received adjuvant chemotherapies, PD-L1+/ high NLR was identified to be associated with worse DFS (log-rank χ^2^=4.582, *P*=0.032) but not OS (log-rank χ^2^=0.120, *P*=0.729).

## Discussion

In the present study, PD-L1 protein expression was elevated in 34.0% of patients with NSCLC who underwent surgery (41.0% of lung SCC samples and 28.5% of lung AD samples). PD-L1-positive expression was more frequently observed in male, heavy smokers and patients with SCC. Multivariate analysis revealed that smoking index ≥400 was independent predictor of PD-L1 expression. No significant correlations between PD-L1 protein expression and NLR or EGFR mutation status had been found. Patients with pretreatment NLR > 2.3 and PD-L1 expression was associated with inferior DFS and OS. Moreover, we demonstrated that such poor prognosis was only observable in cases without EGFR mutations, and the prognostic effect for NLR/PD-L1 might be affected by adjuvant therapy and subsequent treatment.

In previous reports, clinical factors, such as smoking history, were reported to be associated with the PD-L1 expression. Wu et al. reported that PD-L1 protein expression is higher in men than women, smokers than never smokers 20. Takada et al. demonstrated that PD-L1 positivity was significantly associated with male sex, smoking and squamous cell carcinoma 21.Another study showed the high-PD-L1-expression group had a significantly higher proportion of smokers compared with the low-expression group 22. These data are consistent with the results of our study.

Previous researches have also shown that PD-L1 protein expression is associated with EGFR mutations. Takada et al. pointed out that PD-L1 positivity was significantly associated with wild-type EGFR 23. On the contrary, other studies showed that PD-L1 expression was significantly associated with the presence of EGFR mutations 24, 25, which was not observed in our study. The reasons for these discrepancies may be due to the admixture of AD and SCC in our analysis as well as different antibodies and cutoffs. Although our study have found no significant correlations between PD-L1 protein expression and EGFR mutation status, our stratified analysis showed that the prognosis value of combined PD-L1/NLR could only be observed in wild-type patients but not EGFR-mutated patients.

Many studies have evaluated the prognostic impact of PD-L1 protein expression in NSCLC, including the present study. However, the results of these studies vary: some studies have shown that expression of PD-L1 was correlated with poor clinical outcomes in NSCLC^26^, ^27^; while others showed a favorable prognosis for NSCLC with PD-L1 expression^28^, ^29^.In our study, however, no significant correlations between PD-L1 protein expression and prognosis were found, when we analyzed the survival in strata of histology, still no association was found between PD-L1 protein expression and DFS or OS (data not shown). As described above, different antibodies and cutoffs may account for some of the discrepancies.

There are a number of well-established systemic inflammation-based prognostic scores for patients with NSCLC 9. NLR, which reflects alterations in peripheral blood leukocytes associated with systemic inflammatory response, has been studied extensively as a marker of tumor outcomes 5-8, 10-12. Moreover, in the era of immunotherapy, studies have shown that systemic inflammation markers are associated with the outcome of NSCLC patients that treated with nivolumab. Elevated baseline NLR has also been associated with worse outcomes in patients with NSCLC treated with nivolumab^13^. Data from the Swiss has also suggested that pre-treatment NLR was associated with shorter OS and PFS and with lower response rate in patients with metastatic NSCLC treated with nivolumab^14^.Our analyses revealed that, patients with pretreatment NLR>2.3 and PD-L1 expression had significantly shorter DFS and OS, suggesting a possible benefit population with immune checkpoint inhibitors. Further studies are needed to determine whether combination of PD-L1 expression and NLR are predictive or prognostic in NSCLC patients treated with immune checkpoint inhibitors.

Mechanism of regulation of PD-L1 expression has not yet been sufficiently clarified. Previous reports often focused on oncogene-driven mechanisms 30, 31, while a recent study indicated that a distinct, inducible mechanism was operative for PD-L1 expression. Specifically, IFN-γ secreted by TILs promotes PD-L1 expression by tumors and other cells in the immediate tumor microenvironment, which in turn leads to dysregulation of T-cell effector functions via inhibitory PD-1 interaction 32. Inflammation-induced PD-L1 expression by IFN-γ differs from oncogene-induced PD-L1 expression in that PD-L1 expression depends on the time and site of the immune response. We therefore investigated the association between PD-L1 expression and NLR, and whether or not combination of PD-L1 expression and NLR in resectable NSCLC patients can be a prognostic indicator. Our result showed that patients with PD-L1expression and high NLR tend to show poor outcomes. Therefore, we could since speculate that in cases with poor outcomes included those that both raised NLR and broad PD-L1 expression, inflammation-induced PD-L1 expression may be dominant, and an immunosuppressive state in relation to over-production of neutrophils may have occurred, and at the same time, PD-1-mediated tumor immune escape by which the cancer cells can become progressive may have also been activated.

The validation of PD-L1 expression and inflammatory markers may be significant because they offer the potential for a feasible test that can be used to help evaluate the patient's prognosis. However, the tumor-host immune and inflammatory response is a complex interaction, with the key processes underlying this response still unclear. Our study has several limitations. First, the current study included patients with only operable NSCLC, and these patients were not treated with PD-1 or PD-L1 axis therapies. Further studies in patients treated with PD-1 axis therapies should be performed to confirm the findings obtained in the current study. Second, the PD-L1 analysis for surgically resected NSCLC was conducted using a specific antibody against PD-L1 (E1L3N) and with one cut-off value. Immunohistochemical staining of PD-L1 had been performed using different PD-L1 antibody clones in the different studies and clinical trials^33^-^37^, the distribution of PD-L1 expression at the 1% and 50% cut points closely reflected the percentages of the population considered positive in the Keynote^34^ and CheckMate^35^ studies. Many studies compare the commercially available clones. It was shown that the antibodies, from the perspective of interaction with the PD-L1 epitope, are most likely only subtly different 38-42. PD-L1 staining using clone E1L3N has been shown to render equivalent results to antibodies 22C3 and 28-8 in previous studies 38, 43. Parra et al 40 compared a large number of different PD-L1 commercial clones, showed these antibody clones are comparable and presented the feasibility of an equally high sensitivity of PD-L1 staining using clone E1L3N. Therefore, we consider our PD-L1 staining using E1L3N as valid and reliable.

## Conclusion

Taken together, our findings indicate that combination of PD-L1 expression and NLR may be a promising prognostic indicator, and may also be a good marker for tumor recurrence. However, to suggest potential intervention strategies aimed at cancer-associated inflammation so as to improve outcome of immune checkpoint blockade, further study of these mechanisms, including research from a molecular biological approach, and covering more advanced NSCLC cases, is required.

## Figures and Tables

**Table 1:** Correlation between PD-L1 expression and clinicopathological characteristics

Variables	N	χ^2^	PD-L1 expression (%) Negative Positive *P*-value		
**Age (years)**					
< 60	124	1.349	86(69.3)	38(30.6)	0.245
≥ 60	111		69(62.2)	42(37.8)	
**Gender**					
Male	153	5.226	93(60.8)	60(39.2)	0.030
Female	82		62(75.6)	20(24.4)	
**Smoking Index**					
<400	142	18.650	109(76.8)	33(23.2)	<0.001
≥400	93		46(49.5)	47(50.5)	
**Clinical stage**					
I	112	5.097	82(73.2)	30(26.8)	0.078
II	45		26(57.8)	19(42.2)	
IIIA	78		47(60.3)	31(39.7)	
**Histology**					
AD	130	4.036	93(71.5)	37(28.5)	0.045
SCC	105		62(59.0)	43(41.0)	
**EGFR mutation**					
No	187	0.210	122(65.2)	65(34.8)	0.647
Yes	48		33(68.8)	15(31.3)	
**NLR**					
<2.3	155	0.005	102(65.8)	53(34.2)	0.946
≥2.3	80		53(66.3)	27(33.8)	

SCC: squamous cell carcinoma. AD: adenocarcinoma. Smoking index = (number of cigarettes per day) × (duration in years). *P*-values <0.05 in bold.

**Table 2:** Predictive and prognostic values of variables by univariate analysis

Variables	N	DFS			OS		
		Median DFS	χ^2^	*P*-value	Median OS	χ^2^	*P*-value
All patients							
Age (years)							
< 60	124	32.4	0.680	0.410	43.6	0.923	0.337
≥ 60	111	21.0			42.6		
**Gender**							
Male	153	32.4	0.463	0.496	44.0	0.001	0.971
Female	82	23.9			38.6		
**Smoking Index**							
<400	142	30.9	1.153	0.283	51.7	4.832	**0.028**
≥400	93	20.7			35.5		
**Clinical stage**							
I	112	37.4	7.876	**0.019**	54.7	7.582	**0.023**
II	45	32.4			41.2		
IIIA	78	16.2			35.5		
**Histology**							
AD	130	25.5	1.045	0.307	43.7	0.130	0.719
SCC	105	32.4			41.2		
**Adjuvant treatment**							
No	147	21.0	0.371	0.543	36.5	5.440	**0.020**
Yes	88	36.5			52.9		
**EGFR mutation**							
No	187	21.3	0.730	0.393	38.1	2.798	0.094
Yes	48	37.5			54.7		
**PD-L1**							
Negative	155	30.0	1.166	0.280	43.4	0.667	0.414
Positive	80	24.0			41.2		
**NLR**							
<2.3	155	35.0	3.366	0.067	44.0	1.624	0.202
≥2.3	80	20.4			38.1		
**PD-L1 and NLR**							
PD-L1+/NLR _high_	27	16.2	5.231	**0.022**	24.9	4.742	**0.029**
Others	208	30.0			43.7		
**Patients with EGFR wild type**							
PD-L1+/NLR_ high_	23	14.4	5.593	**0.018**	21.9	9.323	**0.002**
Others	164	23.3			41.6		
**Patients with EGFR mutation**							
PD-L1+/NLR_ high_	4	34.7	0.003	0.959	62.3	1.794	0.180
Others	44	38.6			53.8		
**Patients with adjuvant chemotherapy**							
PD-L1+/NLR_ high_	9	24.4	4.582	**0.032**	45.6	0.120	0.729
Others	79	37.5			52.9		
**Patients without adjuvant chemotherapy**							
PD-L1+/NLR_ high_	18	12.0	1.688	0.194	19.3	5.368	**0.021**
Others	129	23.3			38.6		

SCC: squamous cell carcinoma. AD: adenocarcinoma. Smoking index = (number of cigarettes per day) × (duration in years). *P*-values <0.05 in bold.

**Table 3:** Predictive and prognostic values of variables by multivariate analysis

Variables	N	DFS		OS	
		HR (95%CI)	*P*-value	HR (95%CI)	*P*-value
**Clinical stage**					
IIIA	78	1.559(1.123-2.166)	0.009	1.831(1.2749-2.632)	0.001
I- II	157				
**Smoking Index**					
≥400	93			1.616(1.136-2.300)	0.008
<400	142				
**Adjuvant treatment**					
Yes	88			0.544(0.376-0.788)	0.001
No	147				
**PD-L1 and NLR**					
PD-L1+/NLR _high_	27	1.394(1.071-1.811)	0.040	1.442(1.064-1.954)	0.042
Others	208				

HR: hazard ratio. CI: confidence interval. Smoking index = (number of cigarettes per day) × (duration in years).* P*-values <0.05 in bold.
